# BPA and BPS affect the expression of anti-Mullerian hormone (AMH) and its receptor during bovine oocyte maturation and early embryo development

**DOI:** 10.1186/s12958-021-00773-6

**Published:** 2021-08-03

**Authors:** Angela Christina Saleh, Reem Sabry, Gabriela Fabiana Mastromonaco, Laura Alessandra Favetta

**Affiliations:** 1 Department of Biomedical Sciences, Ontario Veterinary College, University of Guelph, Guelph, Ontario Canada; 2 Reproductive Physiology, Toronto Zoo, Scarborough, Ontario Canada

**Keywords:** BPA, BPS, AMH, AMRII, Oocytes, Embryos, Sex ratio

## Abstract

**Background:**

Exposure to endocrine-disrupting chemicals, such as Bisphenol A (BPA) and Bisphenol S (BPS), is widespread and has negative implications on embryonic development. Preliminary evidence revealed that in women undergoing IVF treatment, urinary BPA levels were associated with low serum anti-Mullerian hormone, however a definitive relationship between the two has not yet been characterized.

**Methods:**

This study aimed to evaluate BPA and BPS effects on in vitro oocyte maturation and early preimplantation embryo development through i) analysis of anti-Mullerian hormone (AMH) and anti-Mullerian hormone receptor II (AMHRII), ii) investigation of developmental parameters, such as cleavage, blastocyst rates and developmental arrest, iii) detection of apoptosis and iv) assessment of possible sex ratio skew. An in vitro bovine model was used as a translational model for human early embryonic development. We first assessed AMH and AMHRII levels after bisphenol exposure during oocyte maturation. Zygotes were also analyzed during cleavage and blastocysts stages. Techniques used include in vitro fertilization, quantitative polymerase chain reaction (qPCR), western blotting, TUNEL and immunofluorescence.

**Results:**

Our findings show that BPA significantly decreased cleavage (*p* < 0.001), blastocyst (*p* < 0.005) and overall developmental rates as well as significantly increased embryonic arrest at the 2–4 cell stage (*p* < 0.05). Additionally, both BPA and BPS significantly increased DNA fragmentation in 2–4 cells, 8–16 cells and blastocyst embryos (*p* < 0.05). Furthermore, BPA and BPS alter AMH and AMHRII at the mRNA and protein level in both oocytes and blastocysts. BPA, but not BPS, also significantly skews sex ratios towards female blastocysts (*p* < 0.05).

**Conclusion:**

This study shows that BPA affects AMH and AMHRII expression during oocyte maturation and that BPS exerts its effects to a greater extent after fertilization and therefore may not be a safer alternative to BPA. Our data lay the foundation for future functional studies, such as receptor kinetics, downstream effectors, and promoter activation/inhibition to prove a functional relationship between bisphenols and the AMH signalling system.

## Introduction

The endocrine-disrupting chemical known as bisphenol A (BPA) is an estrogenic compound that is ubiquitous in the environment. BPA is a well-known plasticizer that is commonly used in products such as food and water containers, thermal paper, and the inner lining of aluminum cans [1]. Its pervasiveness was apparent in the recent United States National Health and Nutrition Examination Survey (NHANES) in which BPA was detected in the urine of 92% of study participants [2]. BPA has also been reported in various human biological samples such as blood, perspiration and placental tissue [3, 4].

The most well-known mechanism of action of BPA is its ability to act on both estrogen receptors (ER) alpha and beta, eliciting genomic and non-genomic effects [5, 6]. Current evidence, however, suggests that BPA has a much lower affinity for the ER in comparison to estradiol, thus alternative mechanisms of action to explain its endocrine disrupting function are being explored [5]. As the risk of BPA exposure was revealed, there is an increasing shift towards the use of alternative plastic sources leading to newer and less-researched analogs, such as Bisphenol S (BPS) [7].

BPS, like BPA, is used to form epoxy resins, and is most commonly used in thermal paper such as receipts [8, 9]. Similarly, BPS can also act as an endocrine disruptor altering hormonal function and resulting in various health consequences, especially to the reproductive system. In a preconception cohort assessing men and women seeking fertility evaluation, higher urinary BPA levels were associated with a reduced probability of implantation, clinical pregnancy and live birth, as well as a decrease in antral follicle count [10]. In a variety of species, both BPA and BPS exposure have been linked to increased oxidative stress, impaired oocyte maturation and altered organ weights [7, 11]. BPA and BPS treatment of in vitro matured bovine and porcine oocytes displayed BPA’s ability to disrupt oocyte spindle formation, cumulus cell expansion, meiotic progression and gap junctions’ proteins [12–14]. It has been proven that BPA and BPS can alter oocyte maturation and embryo development outcomes, however, the mechanisms through which this occurs have not yet been elucidated.

Epidemiological studies in fertility clinics have shown that women with higher levels of urinary BPA also exhibited a decreased antral follicle count, indicative of a smaller ovarian reserve and consequently lower ability to conceive [15]. As the anti-Müllerian hormone (AMH), a member of the TGF-ß family, is a gold-standard marker of ovarian reserve and linked to the number of maturing follicles, we could speculate that BPA and its analog, BPS, may have an effect on oocytes maturation, in part, through AMH mediated pathways [15]. It has been shown that BPA exposure can alter other TGF-ß family members that are active within the oocyte, such as reducing BMP-15 expression within oocytes, decreasing the phosphorylation of the TGF-ß downstream target SMAD-3 and increasing the expression of the TGF-ß inhibitor SnoN in ovarian cells [16].

In order to evaluate BPA/BPS effects in vitro, revelant experimental doses must be determined by considering the standardized lowest dose that would result in an adverse effect, also known as the lowest observed adverse effect level (LOAEL). The in vivo LOAEL for BPA is reported to be 50 mg/kg/day and extrapolated to an in vitro 0.05 mg/mL dose in mice [17]. We previously performed dose dependent studies on BPA and BPS, using the specified dose in mice as a reference. Doses 100X and 10X lower as well as 10X higher than the LOAEL were examined and compared to the LOAEL effects on developmental rates in the bovine model, showing that the most environmentally and physiologically significant dose to use in in vitro experiments during bovine early pre-implantation embryo development is the LOAEL dose of 0.05 mg/mL [18].

In many significant aspects of reproductive physiology and embryo development, the bovine and human systems share important similarities. The stages of ovarian follicle development, oocyte maturation and early embryo development, including cleavage of blastomeres, timing of epigenetic reprogramming and maternal to embryonic transition are closely paralleled in cattle and humans [19]. Due to these analogies, as well as the likeness in endocrine function between the species, various studies have shown that the bovine model is reliable for assessing chemical toxicity in human oocytes and in early embryos [20, 21]. Based on these fundamental analogies, we used the bovine system for our experiments as a translational model for human preimplantation development.

Due to the scarcity of knowledge on the mechanisms by which bisphenol compounds disrupt embryonic development, this study aims to further investigate the effects of BPA and BPS on embryo developmental capability. Specifically, this paper intends to highlight and characterize a relationship between these bisphenols and AMH expression at the oocyte and embryo levels, creating a solid foundation for further functional studies. The outcomes may allow us to establish a link between EDCs, oocyte competency and proper early embryonic development and a possible AMH-linked mechanism used by bisphenols during early stages of reproduction.

## Materials and methods

### Oocyte aspiration and in vitro maturation

Bovine (*Bos taurus*) ovaries from a local abattoir (Cargill Meat Solutions, Guelph, ON, Canada) were aspirated to retrieve cumulus oocyte complexes (COCs) into collection medium, consisting of 1 M HEPES 52 (Sigma Aldrich, Oakville, Canada; H3375)-buffered Ham’s F-10 (Sigma Aldrich N6635) supplemented with 2% (v/v) steer serum (Cansera International Inc., Etobicoke, Canada), heparin (2 IU/mL) (Sigma Aldrich H3149), sodium bicarbonate, and penicillin/streptomycin (1%) (Gibco, 15,140–122). Oocyte maturation was carried out using the protocol previously established in our lab [18]. In brief, COCs were collected and washed in serum in vitro maturation medium (S-IVM) consisting of HEPES buffered TCM199 (Sigma Aldrich M4530) supplemented with 2% steer serum and sodium pyruvate (Sigma Aldrich P4562). Oocytes with a dark cytoplasm and tightly packed cumulus cells were selected, washed in S-IVM, and randomly divided between the four treatments, in groups of 60 COCs.

Each group of COCs was washed twice in S-IVM supplemented with hormones (S-IVM + H) prepared as follows: 10 μL of LH (1 μg/mL – NIH, California, United States; AP117438), 12.6 μL of FSH (0.5 μg/mL - Folltropin V; Vetoquinol, Quebec, Canada; 00867357), 10 μL of Estradiol (1 μg/mL – Sigma Aldrich E2785), and 800 μL of Fetal Bovine Serum (FBS) (10% total serum - Gibco, Whitby, Canada 12,483–020) into 10 mLs of S-IVM. The groups were then washed twice in S-IVM + H with the treatments before being placed into 80 μL micro-drops of S-IVM + H. Treatment groups were prepared using 2.5 ml of S-IVM + H for each group: no further additives (control), 2.5 μl of 0.1% ethanol (vehicle), 2.5 μl of BPA (Sigma Aldrich 239,658) and 2.5 μl of BPS (Sigma Aldrich 43,034) dissolved in 0.1% ethanol for a final concentration of 0.05 mg/mL.

Sixty COCs were placed into the micro-drops of the S-IVM + H + treatment with 15–20 COCs per micro-drop. They were then covered with mineral oil (Sigma Aldrich M5310) and placed in an incubator at 38.5 °C in 5% CO_2_ to mature for 24 h. After maturation, 30 COCs were removed and washed 3X in sterile phosphate buffered saline (PBS) (Multicell, Wisent Bioproducts, Quebec, Canada; 311–010) with 0.01% polyvinyl alcohol (PVA) (Sigma Aldrich P8136) and snap-frozen in liquid nitrogen. The remaining 30 COCs were transferred into 500 μL of hyaluronidase (2 mg/mL) from Bovine Testes (Sigma; H3506-1G) for enzymatic and mechanical removal (via micropipette) of the cumulus cells. Oocytes were then washed 3X in PBS + 0.01% PVA and snap-frozen in liquid nitrogen. The remaining cumulus cells were immediately removed from the hyaluronidase and pelleted with PBS + 0.01% PVA, washed in PBS + 0.01% PVA to ensure removal of hyaluronidase and snap-frozen in liquid nitrogen.

### In vitro embryo production

After maturation, COCs were washed twice in HEPES with Bovine Serum Albumin (BSA) (Sigma Aldrich A8806) and twice in BSA-supplemented IVF TALP (Tyrode albumin lactate pyruvate). COCs were then placed in 80 μL droplets of IVF-TALP + BSA covered with mineral oil in an incubator at 38.5 °C and 5% CO_2_. *Bos taurus* semen (Semex, Guelph, ON) was thawed and assessed for motility. The swim-up protocol was performed in 1.5 mL of HEPES Sperm TALP supplemented with 15% BSA and incubated at 38.5 °C and 5% CO_2_ for 45 min. A concentration of 1 × 10^6^ sperm cells/mL/drop was added into each IVF droplet and incubated at 38.5 °C and 5% CO_2_ in the separate treatment groups for 18 h.

Presumptive zygotes (PZs) were mechanically stripped of any remaining cumulus cells and sperm, then washed 3X in HEPES Sperm TALP. They were then washed in Synthetic Oviductal Fluid (SOF) medium supplemented with freshly prepared sodium pyruvate, essential and non-essential amino acids (Sigma Aldrich M5550; Sigma Aldrich M7145), Gentamicin (Sigma Aldrich G1272), 15% BSA and 2% FBS. PZs were then placed in 30 μL droplets of the in vitro culture medium (IVC medium) covered with mineral oil in a low oxygen (5% O_2_) incubator until day-8 blastocysts. Cleavage and blastocyst rates were recorded ~ 40 h and 8 days, respectively, post-fertilization.

### Developmental and arrested rates

Developmental rates were measured at key timepoints [22]: cleavage rate at 40–45 h post-fertilization, 2–4 cells between 45 and 50 h post-fertilization, 8–16 cells between 75 and 80 h post-fertilization, and blastocyst rates at Day-8 post-fertilization. All embryos were produced from oocytes matured in the four treatment groups mentioned above: Control, Vehicle, BPA (0.05 mg/mL) and BPS (0.05 mg/mL). The number of embryos arrested at the 2–4 cell stage, the 8–16 cell stage, and the morula stage, were counted on day-8 post-fertilization in order to determine the rates of embryonic arrest at significant developmental stages [23]. All the rates were calculated as a percentage over the number of cleaved zygotes. Zygotes were considered cleaved if 2 distinct blastomeres were present.

### TUNEL

Terminal deoxynucleotidyl transferase dUTP nick end labeling (TUNEL) was performed using the In Situ Cell Death Detection Kit, Fluorescein (Roche; 11,684,795,910) following the Manufacturer’s instructions in order to detect apoptosis. For each embryo, ImageJ software was used to count the number of cells by detection of the number of stained nuclei (blue). The number of cells with apoptosis was counted manually based on observation of the TUNEL label (green) and apoptosis rates were taken as a ratio of the number of apoptotic cells over the total number of cells. Positive controls were not quantified, they were observed exclusively to assure the success of the technique in each run.

After 8 days of culture in a low oxygen incubator (5% O_2_), blastocysts and arrested embryos were washed 3X in 0.5 mL of PBS with 1% PVA (PBS/PVA) and then fixed in 4% PFA for 20 min at RT and stored in 1% PFA in PBS until used. Embryos were washed 3X in 0.5 mL of PBS/PVA and permeabilized in 0.5 mL of Triton X solution (0.5% Triton X in 1X PBS) for 1 h at RT. After 40 min, the embryos used for the positive control were removed, washed twice in 0.5 mL of PBS/PVA and incubated in a solution of DNase I (Thermo Fisher Scientific, EN0521) for the remaining 20 min in a dark, humidified chamber at 37–38 °C.

After 1 h, all embryos were washed twice in 0.5 mL of PBS/PVA, except for the positive controls which remained in the DNase I. Then all embryos, including the positive controls, were transferred to a micro-well dish, with each well containing 10 μL of the TUNEL reaction mixture (1-part enzyme + 9-parts FITC label). The negative control embryos were placed in a well containing 10 μL of the FITC label only, without the TUNEL enzyme. Embryos were incubated in the TUNEL solution for 1 h in a dark, humidified chamber at 37–38 °C, washed 2X 5 min in 10 μL of Triton X solution, 1X 5 min in 10 μL of PBS/PVA, 1X in 10 μL of RNase buffer at RT and incubated into 10 μL of RNase A (Thermo Fisher Scientific; EN0531) solution for 1 h in a dark, humidified chamber at 37–38 °C in order to digest nuclear RNA. Embryos were then washed twice in 10 μL of RNase buffer, incubated in 10 μL of Hoechst stain solution for 30 min in a dark, humidified chamber at 37–38 °C and mounted onto slides using the same protocol mentioned above and imaged using an Olympus FV1200 Confocal Microscope at 20X or 40X objectives with laser wavelengths of 405 nm for Hoechst (blue) and 488 nm for the TUNEL FITC label (green) using the Fluoview software.

### RNA extraction and reverse transcription

Total RNA was extracted from the frozen groups of either 30 COCs, 30 denuded oocytes, or cumulus cells corresponding to 30 oocytes using the Qiagen RNeasy Plus Micro Kit (Qiagen, Toronto, ON) with a modified protocol. To each sample, 350 μL of Buffer RLT Plus was added, samples were then transferred to a gDNA Eliminator spin column placed in a 2 mL collection tube, centrifuged for 30 s at 10,000 RPM and then 350 μL of 70% ethanol was added to the flow-through. Samples were transferred to a RNeasy MinElute spin column and centrifuged for 15 s at 10,000 RPM. After washes with buffers and 80% ethanol, each column was centrifuged at 13,000 RPM for 5 min to dry the membrane, transferred to a new 1.5 mL collection tube and 17 μL of RNase-free water was added directly to the center of the spin column membrane, followed by 1 min centrifugation at 13,000 RPM to elute the RNA.

To assure reproducibility, all samples were reversed transcribed consistently in groups of 30. RNA was reverse transcribed into cDNA by adding 4 μL of QuantaBio qScript cDNA SuperMix (VWR, Mississauga, Canada; 95,048) to the 16 μL of RNA eluted for each sample (T100 Thermal Cycler, BioRad, Mississauga, ON) and incubated as previously described [18]. Samples were stored at − 20 °C until processed. Additionally, a group of 300 oocytes was reverse transcribed and diluted to a concentration of 3 oocytes/μL and used as a calibrator and for standard curves.

### Quantitative polymerase chain reaction (qPCR)

qPCR was used to measure AMH and AMHRII mRNA expression profiles using a CFX96 Touch Real-Time PCR Detection System (BioRad). Four biological replicates, consisting each of either 30 COCs, 30 denuded oocytes or equivalent stripped cumulus cells, were analyzed for each target and reference gene listed in Table 1. Glyceradehyde-3-phosphate dehydrogenase (GAPDH) and peptidylprolyl isomerase A (PPIA) were used as reference genes based on previous studies performed in our laboratory showing that these reference genes are not affected by the treatments [25]. Relative quantification analysis was performed with a use of Standard curve for each set of primers, by the ΔΔCt method and a calibrator was run in each plate to account for interrun variations.

**Table 1:** qPCR primers

Gene Reference Name	Gene Full Name	Product Size (bp)	GenBank Accession #	Primer Sequence – Forward and reverse (5′-3′)	Efficiency (%)	References
AMH	Anti-Müllerian Hormone	270	NM_173890.1	F: CAGGGAAGAAGTCTTCAGCA R: AAGGTGGTCAAGTCACTCAG	104.4	[24]
AMHRII	Anti-Müllerian Hormone Receptor Type II	163	NM_001205328.1	F: GTGCTTCTCCCAGGTCATAC R: AATGTGGTCATGCTGTAGGC	101.5	[24]
GAPDH	Glyceraldehyde-3-phosphate dehydrogenase	153	NM_001034034.2	F: TTCCTGGTACGACAATGAATTTG R: GGAGATGGGGCAGGACTC	99.5	[25]
PPIA	Peptidylpropyl isomerase A	111	NM_178320.2	F: TCTTGTCCATGGCAAATGCTG R: TTTCACCTTGCCAAAGTACCAC	103.7	[25]
DDX3Y	DEAD (Asp-Glu-Ala-Asp) box polypeptide 3, Y-linked	225	NM_001172595.1	F: GGACGTGTAGGAAACCTTGG R: GCCAGAACTGCTACTTTGTCG	101.5	[26]
USP9Y	Ubiquitin-specific peptidase 9, Y-linked	285	NM_001145509.1	F: GCCAGATGACCAAGAAGCCCCA R: GGACTGTAAGGCCTAATAGCCTGGT	101.1	[26]

Each mRNA target was amplified using SsoFast EvaGreen supermix (Biorad 1,725,201) with 5 μL of EvaGreen, 2 μL of RNase-free water and 1 μL of a working dilution of the forward and reverse primers (25 μM) to a final volume of 8 μL. 2 μL of each cDNA sample was added and reactions performed at: 95 °C for 5 min, followed by 44 cycles of denaturation at 95 °C for 10 s, annealing at 60 °C for 10 s and elongation at 72 °C for 10 s and acquisition of fluorescence at 95 °C for 10 s, ending with the melt curve acquisition from 72 to 95 °C by 0.5 °C increments.

### Western blotting

Quantification of AMH and AMHRII protein in 30 COCs, denuded oocytes and corresponding stripped cumulus cells in each of the four treatment groups was performed by western blotting. Results were analyzed using a minimum of 3 biological replicates. β-Actin (Cell Signalling Technology, Whitby, Canada; 4967) was used as a loading control and densitometry performed using the Bio-Rad Image Lab software and analyzed as a ratio to β-actin expression.

Samples were lysed in 20 μL radioimmunoprecipitation assay (RIPA) buffer and 1% (v/v) protease inhibitors (Biotool, Florida, United States; B14001 and B15001), followed by a freeze-thaw cycle in liquid nitrogen, repeated 4 times. Samples were then placed in a water bath sonicator for 30 min followed by centrifugation at 12,000 RPM at 4 °C for 10 min. Equal volumes of 3X reducing buffer with β-mecaptoethanol (Sigma Aldrich M6250) were added to each sample. Polyacrylamide gels (12%) were prepared using Bio-Rad standard gel recipes.

Proteins were heated at 90 °C for 6 min to denature disulfide bonds and reduce tertiary and quaternary proteins prior to use. Samples were run on the 12% gel in an Invitrogen wet transfer western blot apparatus (Invitrogen, Burlington, ON) at 125 V for 2 h. The gel was then placed in a transfer chamber full of cold 1X transfer buffer of Tris, Glycine and water and was run at 25 V for 3 h in order to transfer the protein onto nitrocellulose paper (Biorad 1,620,115). Nitrocellulose blots were washed 2X 10 min in Tris buffered saline pH 7.6 with 0.1% Tween 20 (Thermo Fisher Scientific, Whitby, Canada; BP337) (TBST), ponceau stained to ensure protein transfer, blocked for 1 h in 5% skim milk in TBST and incubated with each primary antibody of interest at 4 °C overnight: AMH at 1:500 (Abcam, Cambridge, United States; ab229212) and AMHRII 1:2000 (Abcam; ab197148) dilutions, respectively.

After 3X washes in TBST and blots were incubated with the secondary antibody, antirabbit IgG HRP-linked antibody (Cell signaling Technology, Whitby, Canada; 70,745) at 1:5000 dilution for 1 hour, then washed 3X in TBST and incubated with Clarity Western ECL Blotting Substrate (Bio-Rad 170–5060) for 5 min. Blots were imaged on a ChemiDoc XRS + Imaging System (Bio-Rad) and densitometric analysis was used to quantify relative protein expression of AMH and AMHR against the loading control, ß-actin [27].

### Determination of sex ratio via qPCR

qPCR was used to measure DDX3Y and USP9Y mRNA expression profiles using a CFX96 Touch Real-Time PCR Detection System (BioRad). Thirty biological replicates (consisting of individual blastocysts) were processed in each treatment group and GAPDH and PPIA were used as reference genes (Table 1). The same qPCR protocol as for AMH and AMHRII was used.

Previous experiments from our laboratory assessing RNA-based blastocyst sexing, proved that three transcripts are consistently expressed in all male embryos tested and not in females [26]. We selected two of these transcripts, DDX3Y and USP9Y, in order to determine the sex ratio of blastocysts via qPCR. Blastocysts that expressed both of the two exclusively male transcripts selected were classified as male, blastocysts that did not express these transcripts were classified as female and blastocysts that expressed one of the two transcripts were deemed inconclusive and excluded from the results. All samples were run in technical triplicates. RNA was extracted, reverse transcribed and analyzed from a single blastocyst as described previously.

### AMH and AMHRII immunofluorescence detection

Blastocysts were stained with the specific antibody for AMH and AMHRII used for Western Blotting and visualized under confocal microscopy in order to localize and quantify AMH and AMHRII. COCs were used as positive controls as the presence of AMH and AMHRII has been previously confirmed by immunofluorescence in COCs [28]. Semiquantitative analyses was performed using the ImageJ software. On this software each Hoeschst-stained nuclei (blue) were separated and counted, and the mean expression of Alexa Fluor-488 (green) surrounding or within each nucleus were quantified for every blastocyst. An average of the fluorescent levels surrounding or within the nuclei was calculated as the total relative quantification for each blastocyst. Ten biological replicates, consisting each of a single blastocyst in each treatment group, were analyzed for both AMH and AMHRII.

After 8 days of culture, blastocysts were fixed in 4% paraformaldehyde (PFA) for 20 min at room temperature (RT) and then stored in 1% PFA in PBS at 4 °C. Fixed embryos were blocked in 1X PBS supplemented with 0.1% Triton X-100 (Sigma-Aldrich; 9002-93-1) and 5% normal donkey serum (NDS) (Sigma-Aldrich; D9663) for 1 h at RT. Embryos were then washed in 1X PBS for 20 min in a dark, humidified chamber between 37 and 38 °C. Next, embryos were incubated with the primary antibody, either AMH (Abcam ab229212) or AMHRII (Abcam ab197148) at a 1:50 dilution, overnight at 4 °C. Primary antibodies were diluted in antibody dilution buffer containing 1X PBS, 0.005% Triton X and 0.5% NDS. Embryos were washed 3X 30 min in the antibody dilution buffer in a dark, humidified chamber at 37–38 °C and incubated in Donkey anti-Rabbit IgG (H + L) Highly Cross-Adsorbed Secondary Antibody, Alexa-Fluor 488 (Invitrogen, Thermo Fisher Scientific; A21206) at a 1:200 dilution in antibody dilution buffer. Embryos were incubated in the secondary antibody for 1 h in a dark, humidified chamber at 37–38 °C. After 1 h, blastocysts were incubated for 45 min in bisbenzimide H 33258 (Hoechst) nuclear stain (Sigma-Aldrich; B2883-25MG) in a dark, humidified chamber at 37–38 °C. Blastocysts were washed again 3X 30 min in antibody dilution buffer in a dark, humidified chamber at 37–38 °C to decrease background fluorescence.

Blastocysts were then mounted onto slides with Vectashield antifade mounting medium (MJS BioLynx Inc., Brockville, Canada; VECTH1000). Slides were sealed and stored at 4 °C until imaged. Embryos were imaged using an Olympus FV1200 Confocal Microscope at a 40X objective with laser wavelengths of 405 nm for Hoechst (blue) and 488 nm for Alexa-Fluor 488 (green) using the Fluoview software.

### Statistical analysis

Statistical analyses were performed on all data sets using GraphPad Prism 6 and SPSS statistics software. qPCR expression levels were measured and normalized relative to the reference genes as well as to a calibrator using the delta delta Ct method. Western blot protein expression levels were normalized as a ratio to the loading control expression levels. Before assessing statistical differences, each set of data was analyzed for normality using Kolomogorov-Smirnov and Shapiro Wilk tests. If the data were normally distributed, one-way analysis of variance (ANOVA) was used. If the data were not normally distributed, a Kruskal-Wallis test was used. There was a minimum of three biological replicates analyzed per data set. Statistical significance was defined as a *p*-value less than 0.05. Tukey’s post-hoc test was used to compare differences between each treatment group. For embryo sexing data, qPCR expression levels were measured and normalized relative to the reference genes. As blastocyst sex was determined based on the presence or absence of mRNA expression, statistical analyses were performed using a Chi-Squared Post Hoc test for nominal/categorical variables to compare the average number of male and female blastocysts per group.

## Results

### Developmental parameters

**Fig. 1:**
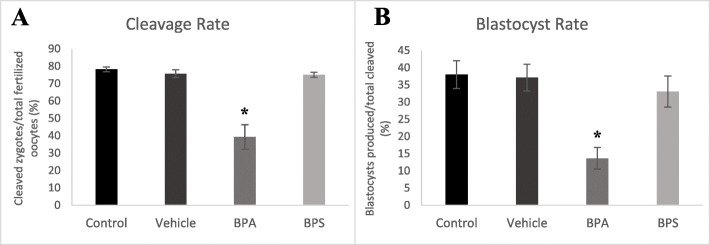
Cleavage and Blastocyst rates. Embryo development following oocyte maturation in each of the four treatment groups (control, vehicle or exposure to either 0.05 mg/mL of BPA or BPS). **A** Percentage of cleaved zygotes at 40–45 h post-fertilization. **B** Percentage of blastocysts developed at day-8 post-fertilization out of total cleaved zygotes. * represents statistical significance *p* < 0.05. Error bars represent +/− SEM (*n* = 6 biological replicates, each representative of one IVF run with 60 starting oocytes per group, each bar represents 360 oocytes)

In vitro matured COCs from each of the treatment groups were cultured to the blastocyst stage and both cleavage and blastocyst rates were recorded. Oocytes exposed to 0.05 mg/mL of BPA for 24 h resulted in a significant decrease in both cleavage (*p* = 0.0001 – Fig. 1a) and blastocyst rates (*p* = 0.0015 – Fig. 1b). No significant effect was observed when oocytes were exposed to the same dose of BPS or to the vehicle alone.

**Fig. 2:**
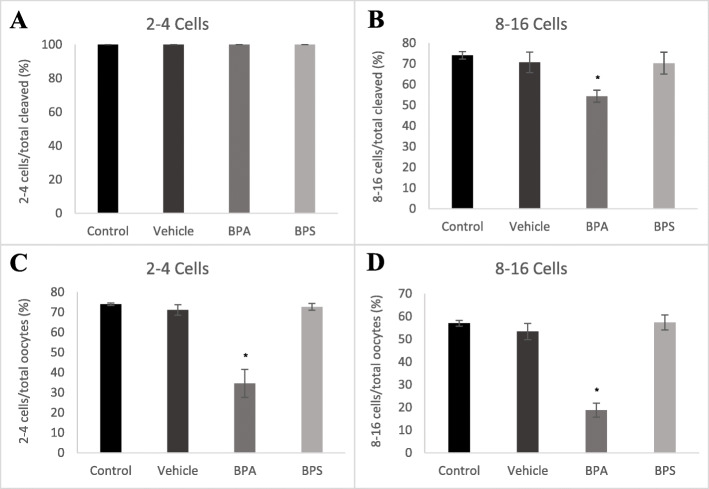
Percentage of arrested embryos at 2–4 cell and 8–16 cell development stages. **A** Embryos that reached the 2–4 cell stages at 45–50 h post-fertilization as a percentage over total cleaved zygotes. **B** Embryos that reached the 8–16 cell stages at 75–80 h post-fertilization as a percentage over total cleaved zygotes. **C** Embryos that reached the 2–4 cell stages at 45–50 h post-fertilization as a percentage over total oocytes. **D** Embryos that reached the 8–16 cell stages at 75–80 h post-fertilization as a percentage over total oocytes. *represents statistical significance p < 0.05. Error bars represent +/− SEM (*n* = 4 biological replicates, each representative of one IVF run with 60 starting oocytes per group, each graph bars represents results from 240 oocytes at the start)

Developmental rates were measured at different stages assessing the number of embryos at the 2–4 cell or at the 8–16 cell stage as a ratio of the number of cleaved zygotes (Fig. 2a and b), or the total number of oocytes (Fig. 2c and d). The number of 2–4 cells at 45–50 h post-fertilization is equivalent to the number cleaved zygotes (Fig. 2a). After BPA exposure during maturation, there was a significantly lower percentage of embryos at the 8–16 cell stage when measured as a ratio to the total cleaved oocytes (*p* = 0.0211) and as a ratio of the total oocytes (*p* = 0.0001) at the 75–80-h post-fertilization time point (Fig. 2b and d). There was also a significant decrease (*p* = 0.0001) in the percentage of 2–4 cells when measured as a ratio to the total number of oocytes (Fig. 2c). No significant effects occurred after BPS exposure.

### Arrested rates

**Fig. 3:**
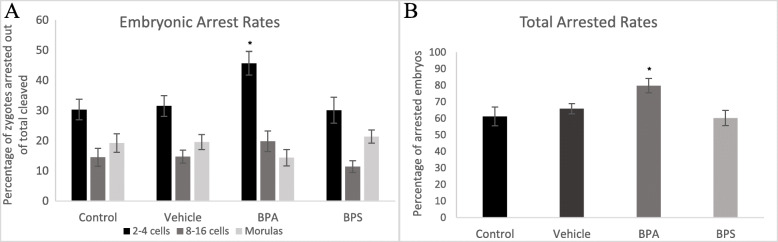
Embryo arrest rates at three developmental stages. **A** 2–4 cells (black), 8–16 cells (dark grey) and morula (light grey). Oocytes were matured in 0.05 mg/mL of either BPA or BPS and fertilized. Arrest rates were counted on day 8 post-fertilization as a percentage of arrested embryos over cleaved embryos. **B** Percentage of total embryonic arrest at day-8 post-fertilization. * represents statistical significance *p* < 0.05 and error bars represent +/− SEM (*n* = 8 biological replicates, each representative of one IVF run with 60 starting oocytes per group)

At eight days post-fertilization, the percentage of embryos arrested at the 2–4 cell stage, 8–16 cell stage and morula stage were calculated. A significantly (*p* = 0.0364) higher percentage of embryos arrested at the 2–4 cell stage when exposed to 0.05 mg/mL of BPA during oocyte maturation (Fig. 3a). There was also a significantly (*p* = 0.0330) higher percentage of total embryonic arrest after BPA exposure (Fig. 3b). These effects were not observed in the BPS treatment group.

### TUNEL

**Fig. 4:**
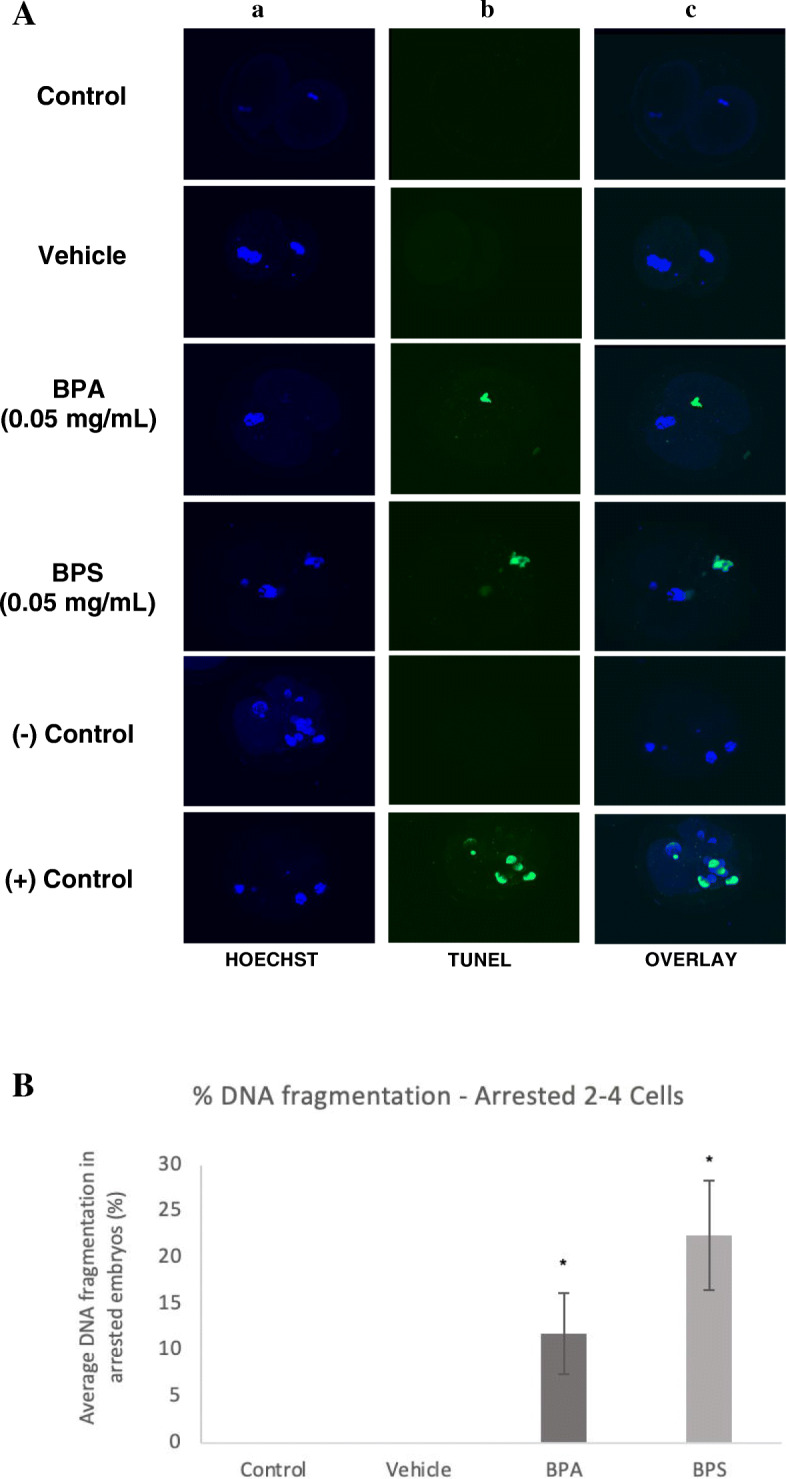
TUNEL assay of DNA fragmentation in arrested 2–4 cells. **A** Column a) is the nuclear counterstain (Hoechst), column b) is the FITC-labelled DNA fragmentation, column c) is an overlay of both. The last two rows display the negative and positive controls of the TUNEL assay. **B** Average DNA fragmentation in arrested 2–4 cells via TUNEL assay. Percentage of DNA fragmentation in each embryo determined as a ratio of the number of cells with DNA fragmentation to the total number of cells in the arrested embryos. * represents statistical significance *p* < 0.05 and error bars represent +/− SEM (*n* = 15 embryos per group)

**Fig. 5:**
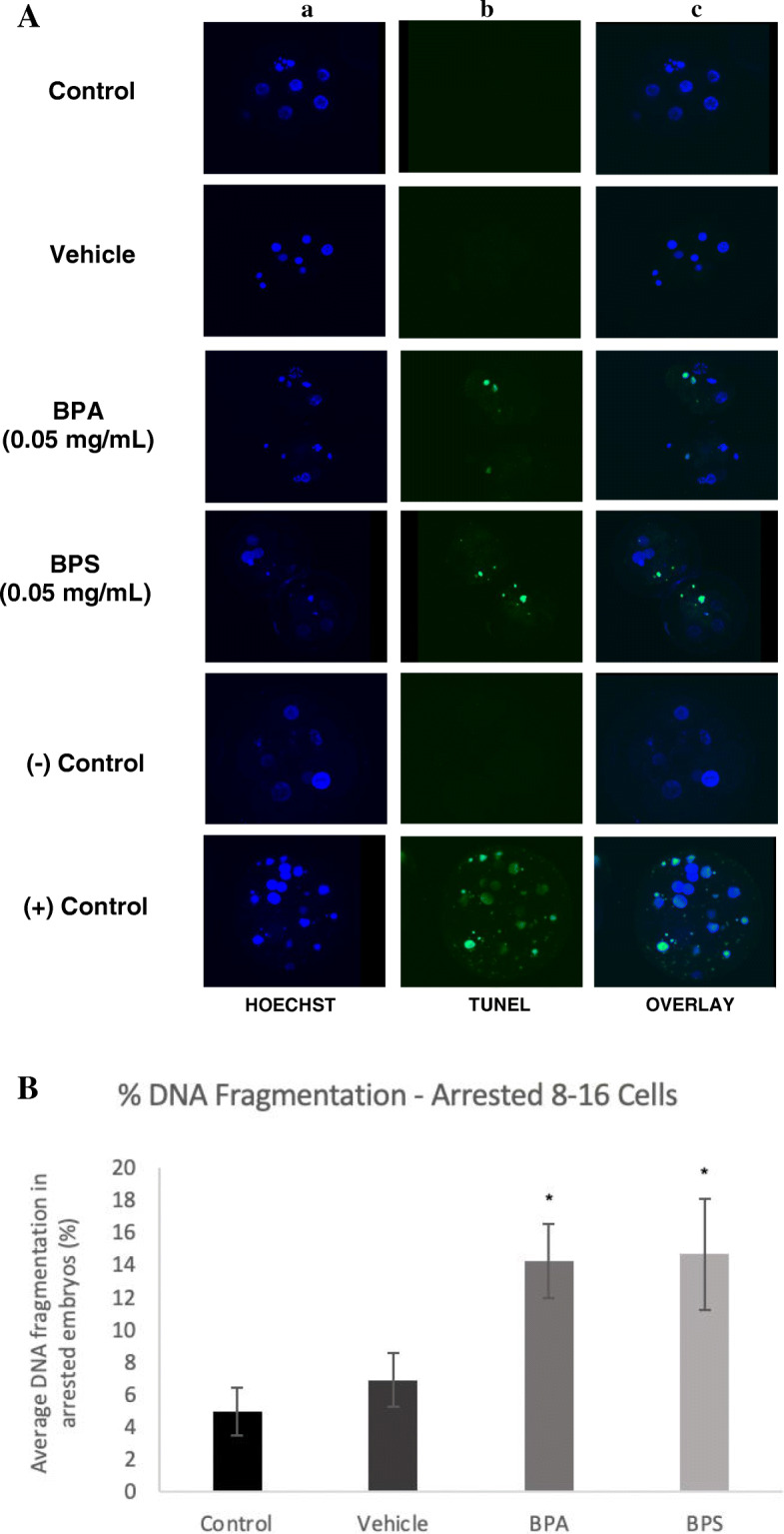
TUNEL assay of DNA fragmentation in arrested 8–16 cells. **A** Column a) is the nuclear counterstain (Hoechst), column b) is the FITC-labelled DNA fragmentation, column c) is an overlay of both. The last two rows display the negative and positive controls of the TUNEL assay. **B** Average DNA fragmentation in arrested 8–16 cells via TUNEL assay. Percentage of DNA fragmentation in each embryo determined as a ratio of the number of cells with DNA fragmentation to the total number of cells in the arrested embryos. * represents statistical significance *p* < 0.05 and error bars represent +/− SEM (*n* = 20 embryos per group)

**Fig. 6:**
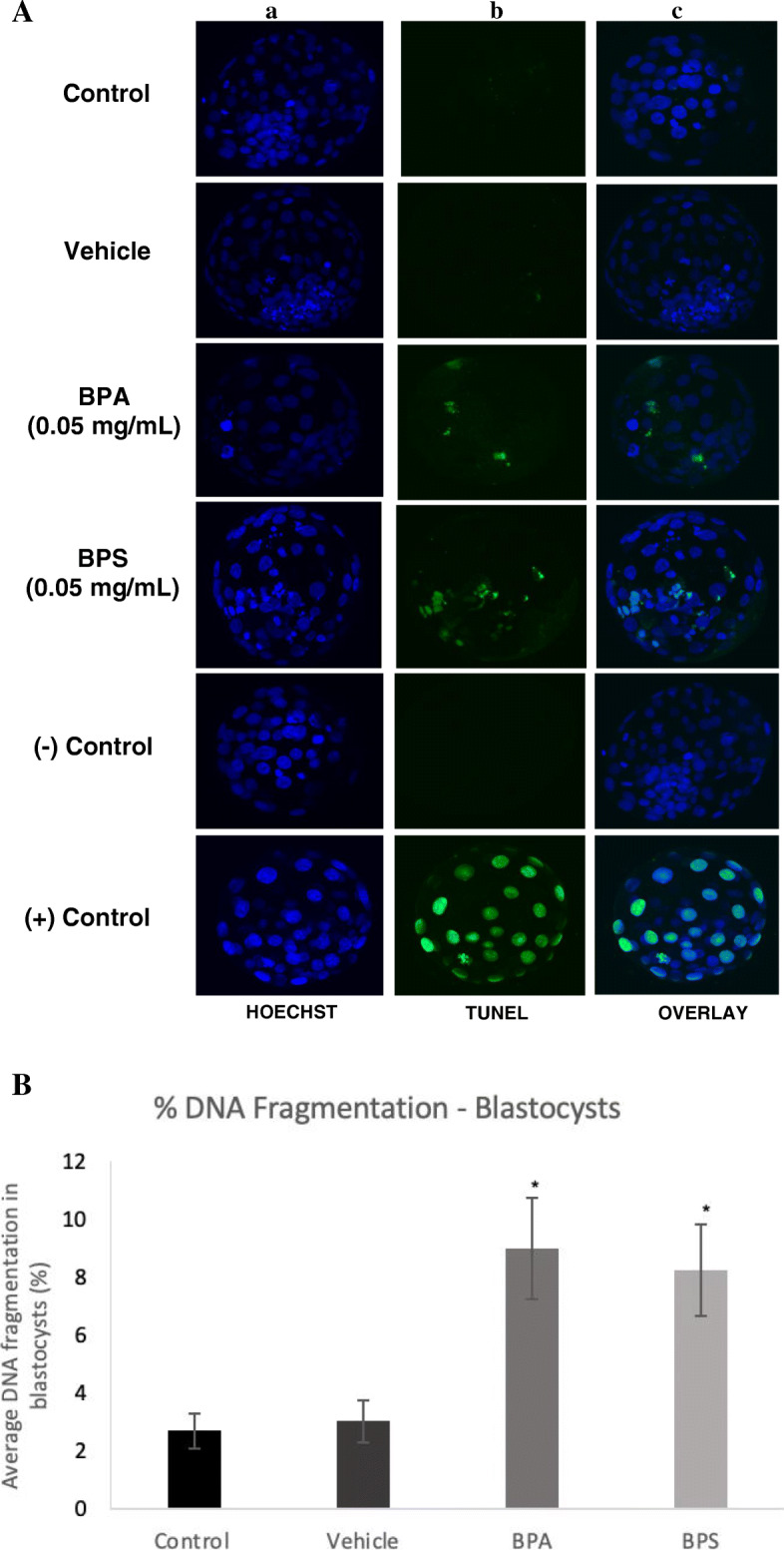
TUNEL assay of DNA fragmentation in day-8 blastocysts. **A** Column a) is the nuclear counterstain (Hoechst), column b) is the FITC-labelled DNA fragmentation, column c) is an overlay of both. The last two rows display the negative and positive controls of the TUNEL assay. **B** Average DNA fragmentation in day-8 blastocysts via TUNEL assay. Percentage of DNA fragmentation in each embryo determined as a ratio of the number of cells with DNA fragmentation to the total number of cells in each blastocyst. * represents statistical significance *p* < 0.05 and error bars represent +/− SEM (*n* = 12 embryos per group)

Terminal deoxynucleotidyl transferase dUTP nick end labeling (TUNEL) was used to measure the presence of DNA fragmentation reflecting the amount of apoptosis in arrested 2–4 cells, arrested 8–16 cells and blastocysts. In arrested 2–4 cells, embryos in the BPA and BPS treatment groups displayed positive staining for DNA fragmentation, while there was no observed apoptosis in the control and vehicle groups (Fig. 4a). There was a statistically significant increase in the average percentage of DNA fragmentation in the BPA and BPS (*p* < 0.01) groups in comparison to the control and vehicle (Fig. 4b). When assessing the arrested 8–16 cell embryos, the TUNEL assay displayed positive staining for DNA fragmentation in embryos exposed to BPA or BPS during oocyte maturation (Fig. 5a). This positive staining in the BPA (*p* = 0.0337) and BPS (*p* = 0.0235) groups was significantly increased in comparison to the control and vehicle groups (Fig. 5b). Figure 6 displays the images of the TUNEL assay performed at the blastocyst stage. The TUNEL assay results observed in the blastocysts were similar to that of arrested 8–16 cells, with baseline levels of apoptosis observed in the control and vehicle groups, and a significantly greater percentage of DNA fragmentation in the BPA (*p* = 0.0058) and BPS (*p* = 0.0176) treatment groups (Fig. 6b).

### AMH and AMHRII mRNA expression in BPA and BPS treated oocytes

**Fig. 7:**
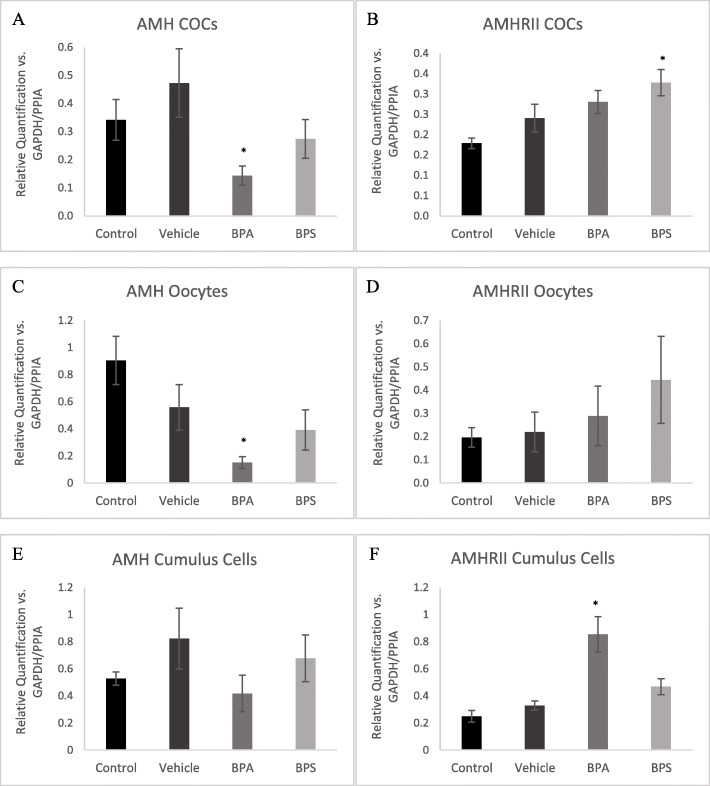
AMH & AMHRII mRNA expression. AMH (**A**) and AMHRII (**B**) in COCs after maturation for 24 h in 0.05 mg/mL of BPA or BPS. AMH (**C**) and AMHRII (**D**) in denuded oocytes after maturation for 24 h in 0.05 mg/mL of BPA or BPS. AMH (**E**) and AMHRII (**F**) in cumulus cells after maturation for 24 h in 0.05 mg/mL of BPA or BPS. Quantification is relative to reference genes GAPDH and PPIA. * indicates statistical significance *p* < 0.01, error bars represent +/− SEM (*n* = 5 biological replicates, each representative of 30 COCs or oocytes or equivalent cumulus cells)

AMH and AMHRII mRNA were quantified using qPCR in COCs, denuded oocytes and cumulus cells relative to reference genes GAPDH and PPIA. In COCs treated with BPA, but not BPS, AMH mRNA expression was significantly reduced (*p* = 0.0277) (Fig. 7a). AMHRII mRNA expression however was significantly increased (*p* = 0.0116) in the BPS treatment group (Fig. 7b). AMH mRNA expression in denuded oocytes was significantly decreased (*p* = 0.0277) in the BPA exposure group (Fig. 7c). There were no significant alterations in AMH mRNA levels in BPA and BPS treated cumulus cells, however, there was a significant increase (*p* = 0.0025) in AMHRII mRNA levels in BPA exposed cumulus cells (Fig. 7f). No statistically significant differences were detected between control and vehicle alone groups.

### Protein expression in BPA and BPS treated oocytes

**Fig. 8:**
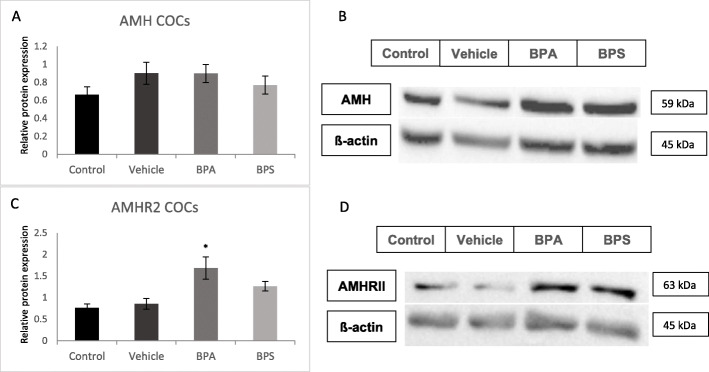
Western blot of AMH and AMHRII protein expression in COCs. **A** Densitometric analyses of AMH expression relative to ß-actin expression. **B** Western blot image of AMH expression and corresponding ß-actin expression. **C** Densitometric analyses of AMHRII expression relative to ß-actin expression. **D** Western blot image of AMHRII expression and corresponding ß-actin expression. *represents statistical significance *p* < 0.05. Error bars represent +/− SEM (*n* = 4 biological replicates, each representative of 30 COCs)

**Fig. 9:**
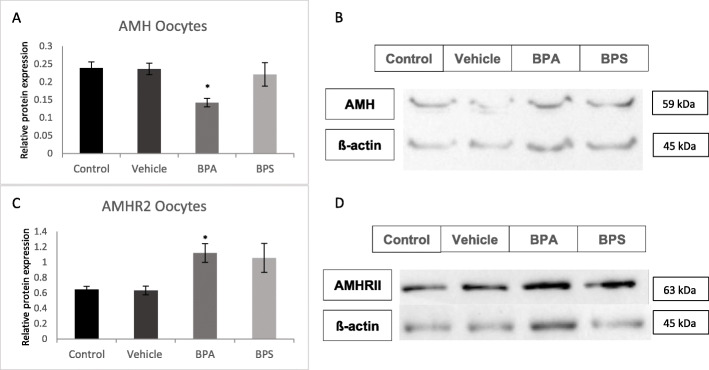
Western blot of AMH and AMHRII protein expression in denuded Oocytes. **A** Densitometric analyses of AMH expression relative to ß-actin expression. **B** Western blot image of AMH expression and corresponding ß-actin expression. **C** Densitometric analyses of AMHRII expression relative to ß-actin expression. **D** Western blot image of AMHRII expression and corresponding ß-actin expression. *represents statistical significance *p* < 0.05. Error bars represent +/− SEM (*n* = 4 biological replicates, each representative of 30 oocytes)

**Fig. 10:**
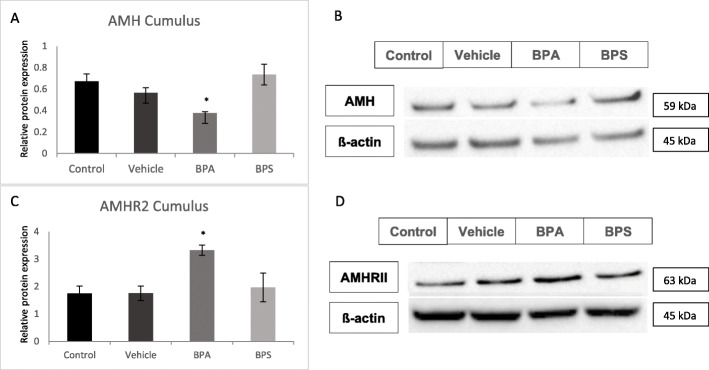
Western blot of AMH and AMHRII protein expression in Cumulus cells. **A** Densitometric analyses of AMH expression relative to ß-actin expression. **B** Western blot image of AMH expression and corresponding ß-actin expression. **C** Densitometric analyses of AMHRII expression relative to ß-actin expression. **D** Western blot image of AMHRII expression and corresponding ß-actin expression. *represents statistical significance *p* < 0.05. Error bars represent +/− SEM (*n* = 3 biological replicates, each representative of cumulus cells equivalent to 30 COCs)

AMH and AMHRII protein levels were quantified in COCs, oocytes and cumulus cells using western blotting relative to the loading control, ß-actin. AMH protein levels were not altered in any of the treatment groups for COCs, however, AMHRII expression was increased in BPA and BPS-treated COCs (Fig. 8c and d) with statistical significance (*p* = 0.0073) reached only in the BPA group. In denuded oocytes, AMH expression was significantly decreased (*p* = 0.0322) and AMHRII expression was significantly increased (*p* = 0.0385) after BPA exposure (Fig. 9). There was a trend towards increased AMHRII expression in denuded oocytes after exposure to BPS, however, this was not statistically significant (*p* = 0.1121). Finally, in cumulus cells there was a significant decrease in AMH protein expression (*p* = 0.0458) and a significant increase in AMHRII expression (*p* = 0.0431) after BPA exposure (Fig. 10).

### Sex ratio

**Fig. 11:**
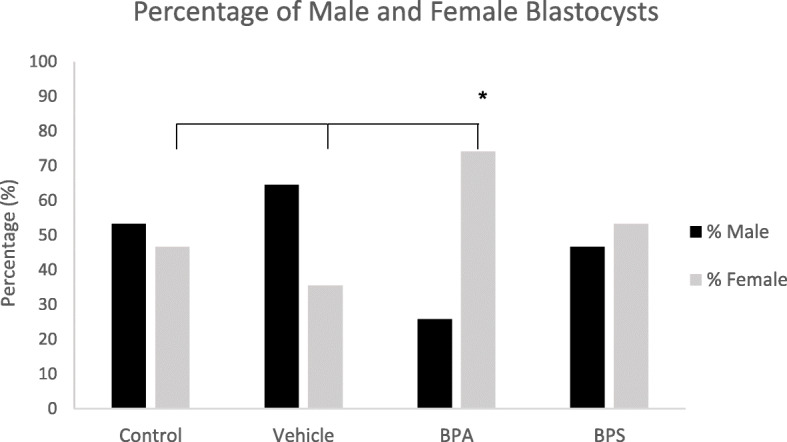
Sex Ratios of treated blastocysts. Percentage of male (black) and female (grey) blastocysts produced from oocytes matured for 24 h in either BPA (0.05 mg/mL) or BPS (0.05 mg/mL). Data were obtained through presence or absence of DDX3Yand USP9Y transcripts detected via qPCR (*n* = 30 blastocysts)

Thirty blastocysts in each treatment group were categorized as either male or female based on the presence or absence of the male transcripts DDX3Y and USP9Y via qPCR. As displayed in Fig. 11, there was about a 50:50 ratio of male to female blastocysts observed in the control and BPS treatment groups. The vehicle group had a slightly greater percentage of blastocysts expressing DDX3Y and USP9Y mRNA, however, this difference was not statistically significant from the control group. Based on chi squared analysis there was a significantly greater number of female blastocysts than male blastocysts in the BPA group. Additionally, there was a significantly higher percentage of females, and lower percentage of males, in the BPA group compared to any other treatment group (Fig. 11). This effect was not observed in the BPS group.

### Immunofluorescence

**Fig. 12:**
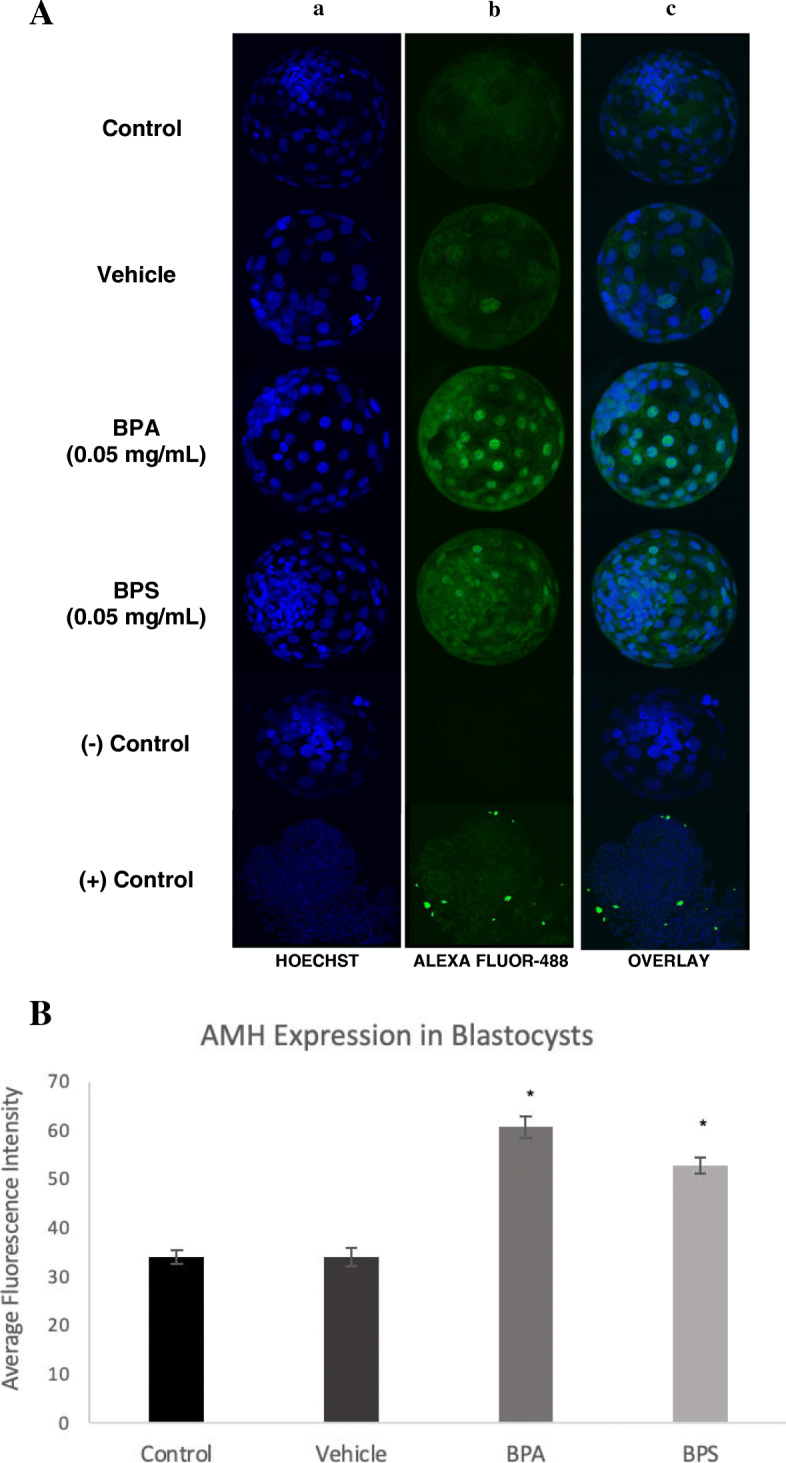
AMH immunofluorescence localization in blastocysts. **A** Blastocysts were fixed and stained with AMH antibody bound by Alexa Fluor-488 conjugated secondary antibody (green). Hoechst stain was used as the nuclei counterstain (blue). Column a) is the nuclear stain, column b) reflects the AMH expression, and column c) is an overlay of both the blue and green channels. The last two rows represent the positive control (bovine COC) and the negative control (blastocysts cultured in Alexa Fluor-488 without primary antibody). **B** Relative quantification of AMH protein levels in blastocysts (*n* = 10) determined by immunofluorescence measure. Bars represent the mean intensity of AMH in each blastocyst determined by intensity of Alexa Fluro 488-conjugated antibody measured with the ImageJ software. * represents statistical significance *p* < 0.01. Error bars indicate +/− SEM (*n* = 10 blastocysts)

**Fig. 13:**
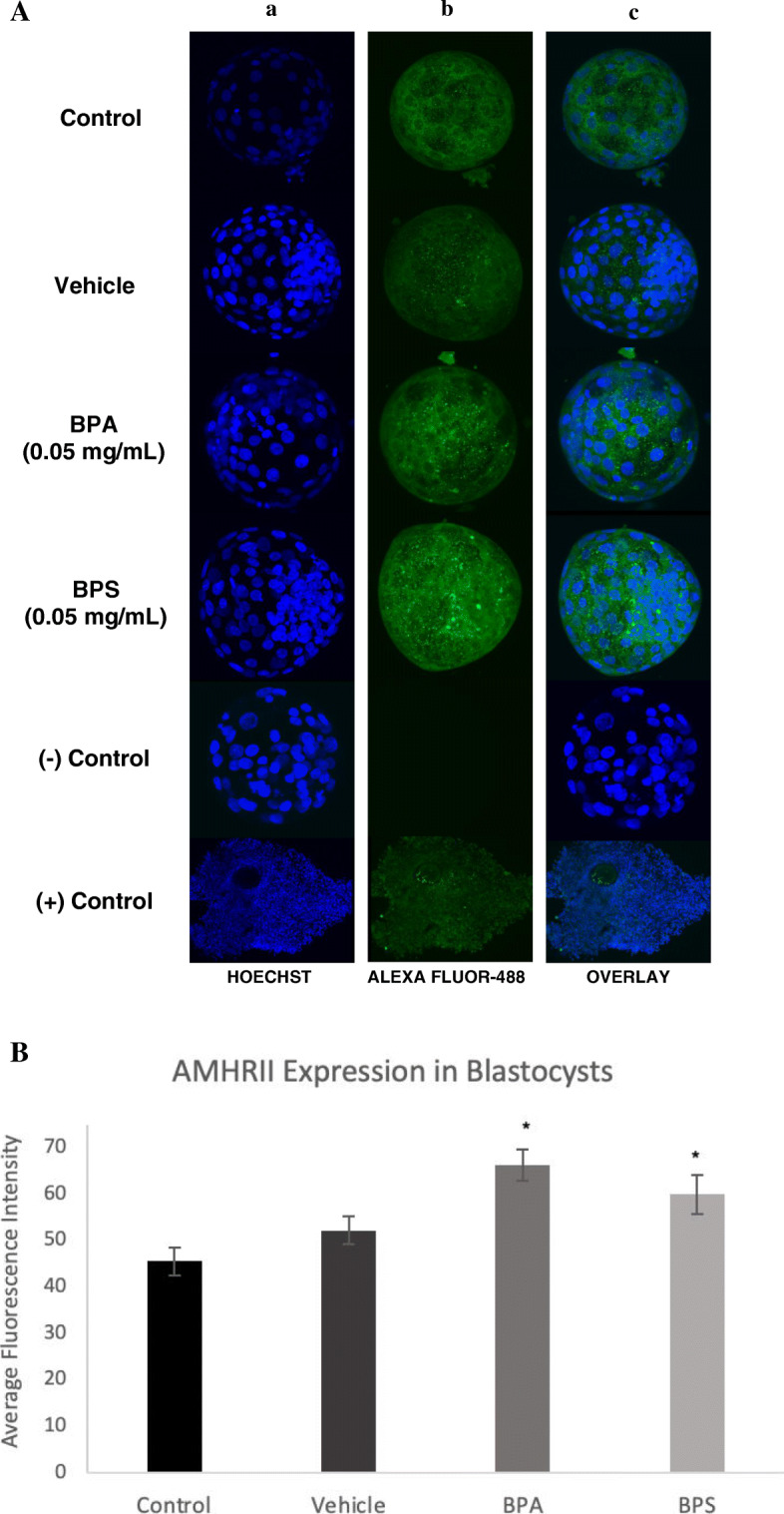
AMHRII levels and localization in blastocysts. **A** Fixed blastocysts were stained with AMHRII antibody bound by Alexa Fluor-488 conjugated secondary antibody (green). Hoechst stain was used as the nuclear counterstain (blue). Column a) is the nuclear stain, column b) reflects AMHRII localization and amount, and column c) is an overlay of both channels. The last two rows represent the positive control (bovine COCs) and the negative control (blastocysts cultured in Alexa Fluor-488 without primary antibody). **B** Relative quantification of AMHRII protein levels in blastocysts (*n* = 10) determined by immunofluorescence. Bars represent the mean intensity of AMHRII in each blastocyst determined by intensity of Alexa Fluro 488-conjugated antibody measured by ImageJ software. * represents statistical significance *p* < 0.05. Error bars represent +/− SEM (*n* = 10 blastocysts)

Protein levels of AMH and AMHRII were quantified as the total immunofluorescence present in each blastocyst using the ImageJ software. Blastocysts were imaged using Olympus FV1200 Confocal Microscope at a 40X objective. Images taken on the Fluoview software reveal AMH expression localized in both the cytoplasm and nucleus of each blastomere (Fig. 12a), while AMHRII expression is solely confined to the cytoplasm, surrounding each of the nuclei (Fig. 13a). Immunofluorescence analysis of the blastocysts showed a significant increase in AMH (*p* = 0.0001) protein expression after BPA and BPS exposure (Fig. 12b) and a significant increase in AMHRII protein expression in the BPA (*p* = 0.0011) and BPS (*p* = 0.0342) treatment groups (Fig. 13b) in comparison to control and vehicle groups.

## Discussion

This study is one of the few reports to explore an association between endocrine disruptors, specifically BPA and BPS, and AMH during early embryonic development. Investigation of the ability of BPA and BPS to induce changes in AMH and AMHRII expression provides potential for uncovering novel mechanisms of bisphenol action during bovine oocyte maturation and early embryo development. Bovine oocytes were exposed to vehicle alone, BPA or BPS during maturation and subsequent analyses of oocyte competence and blastocyst development were performed. These analyses ranged from assessments of cleavage and blastocyst rates, embryonic arrest and apoptosis to quantify AMH and its receptor at the oocyte and blastocyst stages. Significant effects of BPA and BPS were detected, while no statistical significant differences were observed between the vehicle alone and the control group throughout.

Successful embryo development relies heavily on the early stages of folliculogenesis and oogenesis. AMH production by granulosa cells within the follicle allows for communication with the oocyte in order to stimulate growth and maturation [29]. Following oocyte exposure to 0.05 mg/mL of BPA or BPS during maturation, a general decrease in AMH and an increase in AMHRII mRNA and protein levels within the oocytes was observed. From this trend, we can speculate that a compensatory effect is occurring, where AMH is reduced in response to BPA exposure, resulting in an AMHRII increase. Research shows that TGF-ß receptors can be rapidly mobilized to the cell surface from intracellular stores when needed to increase a cell’s responsiveness to the TGF-ß signalling [30]. Therefore, as a member of the TGF-ß family of signalling proteins, AMHRII may have increased expression at the cell surface in order to increase its signalling after a reduction in ligand expression levels.

As there are no definitive findings in literature that characterize BPA-activated AMH pathway, we speculate on possible mechanisms by which BPA increases AMH receptor expression. It is well known that reproductive cells including granulosa cells are hormone sensitive cells that differentially exhibit various receptors at different times of development. During the estrus phase of the bovine cycle, follicular cells begin to display FSH receptors to allow the cells to be responsive to FSH for continued follicle growth. We speculate that AMH receptors are also exhibited during crucial times in development to allow the cells to be AMH sensitive. When AMH levels are high, it may inhibit transcription of more AMH receptors and vice versa when AMH levels are low, this could signal induction of AMHR transcription to compensate for low bioavailability of the AMH ligand.

An alternative possibility is that BPA and/or BPS bind to the AMHRII and stimulate its expression. We observed increases in both AMHRII mRNA levels after BPS exposure in COCs and AMHRII protein levels after BPA exposure in oocytes and cumulus cells. The molecular structures of BPA and BPS are very similar with only the central component differing [9]. The possession of identical phenol groups in both of these chemicals allows them to act on similar receptors and has been linked to their estrogenic abilities [9]. It is plausible that these phenol groups also allow them to bind to AMHRII and induce a response.

While we observed an increase in AMHRII mRNA with BPS exposure of COCs, we did not detect any other significant effects of BPS at the oocyte stage. We observed an increase in AMHRII that trends towards significance after BPS exposure in the denuded oocytes and cumulus cells, however not to the same extent of BPA. Based on our results it appears that BPS may exhibit a reduced potency to BPA in respect to altering AMH and AMHRII expression. It is also important to note that contrary to our observations, most studies have been unable to detect AMH within the oocyte. To our knowledge, this is the first study to detect substantial mRNA and protein levels of AMH in denuded bovine oocytes. Zhang et al. [31] were able to detect AMH mRNA in cumulus cells, but not within the oocyte when assessing mouse oocytes in vitro. They were, however, able to detect substantial levels of AMHRII mRNA within human oocytes. Contrarily, our results obtained both AMH and AMRHII mRNA expression in the oocyte that was in the same range of that found within the COCs and cumulus cells. This discrepancy with the results observed by Zhang et al. [31] may be due to differences between the model species or differences in methodology. While we used qPCR to quantify mRNA expression, the results by Zhang et al. [31] were obtained using reverse-transcription PCR (RT-PCR) and agarose gel, a method shown to be less sensitive and precise in comparison to qPCR [32].

After assessment of BPA and BPS exposure at the oocyte stage, fertilization of these oocytes allowed for subsequent analysis of the downstream effects on embryonic development. In support of previous experiments in our laboratory [25], we observed a significant decrease in both the cleavage and blastocyst rates after BPA exposure, however, this effect was not observed in the BPS treatment group. In addition to decreased cleavage and blastocyst development, embryos in the BPA treatment group also showed an impaired ability to reach the appropriate developmental stages in a timely manner. We observed significant decreases in 2–4 cells and 8–16 cells at the appropriate time points after BPA exposure.

As bisphenol exposure occurred solely during oocyte maturation, we can speculate that these observed outcomes were due to reduction in oocyte competence. These findings suggest that BPA may be delaying, or more likely, inhibiting embryo development. On day-8 of embryo development we also observed a significant increase in embryonic arrest, especially at the 2–4 cell stage, in the BPA treatment group. One of the most commonly reported sources of embryonic arrest is oocyte chromosomal abnormalities and aneuploidy. Exposure of mammalian oocytes to BPA has been shown to result in spindle abnormalities, chromosome misalignments and aneuploidy [12, 33, 34]. Thus, it is feasible that exposure of oocytes to BPA during maturation reduces oocyte competence by impacting chromosomal development, thereby resulting in the observed decrease in cleaved embryos and/or increase in developmental arrest.

Downstream effects of impaired oocyte competence may also manifest through reduction in embryo quality. Sex ratio is a recognised indicator of embryo quality and exposure to environmental stress [35]. Typically, a ratio of approximately 50% males to 50% females is the norm, however variations to this ratio have been observed in altered environmental conditions. Previously, our laboratory observed a skew towards female blastocyst production after oocyte exposure to BPA using qPCR analysis of testis specific protein Y (TSPY) [25]. Using a different analyses, mRNA levels of DDX3Y and USP9Y, two exclusively male transcripts, we detected a comparable same sex ratio skew following BPA exposure as previously reported, however no sex ratio skew was observed following BPS exposure. Whether this skew is the result of BPA increasing the female blastocyst production or simply impairing male blastocyst development has not yet been determined. Notably, this skew has been observed in similar experiments assessing the effects of another endocrine disruptor, dioxin [36]. Not only has dioxin been associated with a skew in embryo sex ratio, but it was also found to significantly reduce viable Y-bearing spermatozoa which was predicted to be the cause of a female-biased sex ratio [37].

As our study exposed the oocytes to BPA before fertilization, we might speculate that the sperm came in contact to BPA and BPS upon entry into the oocyte. Previous ELISA experiments in our laboratory exposing oocytes to BPA have shown that the oocyte takes up 1.69–2.48 ng/mL of BPA [12]. As previously mentioned, BPA has also been detected in follicular fluid and granulosa cells of the follicle [38]. Therefore, spermatozoa exposure to BPA while travelling through the cumulus cells and upon entry into the oocyte is a possibility. This would reduce the viability of the Y-bearing sperm attempting to fertilize the oocytes and likely result in more X-bearing sperm being successful, thus encouraging the skew towards female blastocyst production. Future studies would need to further assess if BPA becomes sequestered in the cytoplasm of an oocyte and if BPA can in fact impact entering spermatozoa in order to support these speculations.

Typically, AMH expression is associated with two major areas of embryonic development; production by granulosa cells to promote growth of ovarian follicles and fetal gonad sexual differentiation [39]. During preimplantation embryo development however, AMH and AMHRII expression have not been characterized. To our knowledge, this is the first study to observe significant AMH and AMHRII expression at the blastocyst stage in bovine embryos. Not only did we detect these proteins in the blastocysts, but they were also significantly increased in BPA and BPS-exposed embryos. Intriguingly, DNA fragmentation was also identically increased in blastocysts from these treatment groups.

Induction of apoptosis may occur through a variety of mechanisms, however, based on our results, we speculate that increased AMH and AMRHII in blastocysts, after BPA and BPS exposure, plays a functional role in promoting apoptosis. One of the most commonly reported roles of AMH during fetal development is pro-apoptotic activity. Through activation of the type II AMH receptor, AMH promotes cell cycle arrest and apoptosis of the Müllerian ducts in the male fetus [40]. In addition to this more conventional method of AMH-induced apoptosis, AMH and its receptor have been associated with apoptosis in other tissues. A study assessing AMHRII expression in granulosa cell tumors found that there was increased AMHRII levels in these tumor cells, which was also positively correlated to caspase-3 expression [41]. This suggests a link between pathological conditions, such as cancer or exposure to endocrine disruptors, and increased AMHRII in order to stimulate apoptosis and removal of damaged cells. If the observed increase in DNA fragmentation is in fact associated with the increased AMH and AMHRII levels, as speculated, future studies should confirm this through antagonism of the AMHRII to observe if the effects are partly mediated by AMHRII signalling. Alternatively, AMH expression in the blastocyst may not only be present for its relation to apoptosis, but also because of AMH being part of the TGF-ß family of glycoproteins. The TGF-ß signalling pathway is involved in cell division, differentiation and apoptosis and it has been suggested that the TGF-ß family, including their downstream effectors, SMAD proteins, are involved in preimplantation embryo development [42].

It is important to note that the AMH promoter contains estrogen-response element (ERE) binding sites [43]. The estrogenic capabilities of BPA and BPS have been well-characterized, and therefore, their ability to induce activation of these estrogenic promoter regions within AMH would explain the increased protein levels observed within the blastocysts. Further research into the presence of a functional role of bisphenols on the AMH promoter would provide insight into AMH transcriptional regulation and how it may be altered by BPA and BPS. It is also important to note that these observed increases in AMH and AMHRII protein as well as DNA fragmentation were effects seen not only after BPA exposure, but after BPS exposure as well. DNA fragmentation in arrested embryos and blastocysts exposed to BPS was also either equal to or greater than that observed in the BPA group. Previous experiments that exposed mice to BPS found that doses greater than 10 μg/kg induced developmental arrest and significantly increases oxidative stress, which is typically associated with apoptosis [44]. Therefore, while BPS had few significant effects at the oocyte stage in comparison to BPA, we observed many significant effects of BPS post-fertilization which may have impaired embryo quality.

Future studies should involve more functional analyses of the interaction between bisphenols and AMHRII. They should also further investigate the role of AMH and AMHRII at the blastocyst stage and the consequences of their altered expression by endocrine disruptors. Our results determined by the TUNEL assay demonstrated the presence of DNA fragmentation in arrested 2–4 cell embryos. While 2–4 cell embryos have been observed to display externalization of phosphatidylserine (PS), an early marker of apoptosis, some evidence suggests that they unlikely possess the necessary machinery for apoptosis [45–47]. Therefore, further analysis is necessary to determine if our observations in the arrested 2–4 cells are in fact DNA fragmentation due to apoptosis or independent from it.

## Conclusions

In summary, exposure of in vitro bovine oocytes to BPA and BPS (0.05 mg/mL) resulted in both short-term (oocyte) and long-term (blastocyst) developmental effects. The implications of exposure to bisphenols through alterations in AMH and AMHRII at the mRNA and protein levels were demonstrated during oocyte maturation. While the majority of significant observations on the oocytes occurred following BPA treatment, we later showed that BPS exposure can have significant downstream consequences on embryo development, suggesting that through different mechanisms both these bisphenols would likely have disruptive effects during early development. Not only were BPA and BPS exposure associated with increased AMH and AMHRII levels at the blastocyst stage, but they were also associated with increased embryonic arrest and apoptosis. Further investigations can build upon the results presented here to outline the mechanisms and aid in the prevention of bisphenol-induced consequences on early embryo development that are associated with AMH/AMHRII signalling.

## Data Availability

The datasets used and/or analysed during the current study are available from the corresponding author on reasonable request.

## Abbreviations

BPA: Bisphenol A
BPS: Bisphenol S
AMH: Anti-Mullerian hormone
AMHRII: Anti-Mullerian hormone receptor II
qPCR: quantitative polymerase chain reaction
NHANES: National Health and Nutrition Examination Survery
ER: Estrogen receptor
LOAEL: Lowest observed adverse effect level
EDCs: Endocrine disrupting compounds
COCs: Cumulus-oocyte-complexes
S-IVM: Synthetic in vitro maturation
FBS: Fetal bovine serum
LH: Luteinizing hormone
FSH: Follicle stimulating hormone
PBS: Phosphate buffered saline
PVA: Polyvinyl alcohol
BSA: Bovine serum albumin
IVF: In vitro fertilization
TALP: Tyrode albumin lactate pyruvate
PZ: Presumptive zygote
SOF: Synthetic oviductal fluid
IVC: In vitro culture
TUNEL: Terminal deoxynucleotidyl transferase dUTP nick end labeling
GAPDH: Glyceraldehyde-3-phosphate dehydrogenase
PPIA: Peptifylprolyl isomerase A
RIPA: radioimmunoprecipitation assay
TBST: Tris buffered saline with tween
ANOVA: Analysis of variance
TSPY: Testis specific protein Y
ELISA: Enzyme linked immunisorbent assay
ERE: Estrogen response element
PS: Phosphotidylserine
